# The efficacy of nalmefene on anesthetic recovery of patients: a study protocol for a multicenter randomized controlled trial

**DOI:** 10.1186/s13063-023-07169-4

**Published:** 2023-03-01

**Authors:** Xiaowen Ma, Jueying Liu, Ying Tang, Qiueyue Lian, Xiaorong Huai, Wanfeng Liu, Diansan Su

**Affiliations:** Department of Anaesthesiology, Shanghai Jiaotong, Renji Hospital, University School of Medicine, Shanghai, China

**Keywords:** Nalmefene, Anesthesia, Recovery quality, Randomized controlled trial

## Abstract

**Introduction:**

So far, the recovery quality after general anesthesia is still unsatisfied. Nalmefene is a drug to treat opioid overdose and reverse opioid actions. We aim to investigate the efficacy of nalmefene on optimizing the recovery quality of patients after general anesthesia.

**Methods:**

It is a prospective, placebo-controlled, two-arm parallel groups, multicentre, double-blind, randomized (PPPMDR) clinical trial. The participants (*n* = 520) will be randomly assigned into two groups. Each patient will receive either: a single dose of nalmefene 0.25 µg/kg in the intervention group, or the same volume of 0.9% NaCl solution in the control group at the end of the surgery. The primary outcome will be the time interval between the end of anaesthesia and recovery endpoints achieved (Aldrete recovery score ≥ 9) in post-anesthesia care unit (PACU). The other variables are the time interval from the end of operation to extubation; Richmond Agitation Sedation Scale (RASS) score at extubation; the time at Montreal Cognitive Assessment Scale (MoCA) orientation score ≥ 5; visual analog scale (VAS) score and adverse effects including postoperative nausea and vomiting (PONV), and pruritus in PACU and 24 h postoperatively.

**Analysis:**

This trial aims to study whether small dose of nalmefene can shorten the time from the end of surgery to Aldrete score ≥ 9 and improve opioid-induced side effects.This trial focuses on providing the reliable clinical evidence for satisfactory quality of recovery.

**Ethics and dissemination:**

This clinical trial has been approved and supported by the ethics committee of the Renji Hospital, Shanghai Jiaotong University, School of Medicine (KY2020-150); Shanghai Tongren Hospital (2021–030-01);The First Affiliated Hospital of Guangxi Medical University (2021–032); and The First Affiliated Hospital of Zhengzhou University(2021-KY-0495–003). Analysis of the study results will be submitted to a peer-reviewed journal for publication.

**Trial registration:**

ClinicalTrials.gov, NCT04713358, Registered on September 23, 2021.

## Introduction

According to the World Health Organization (WHO)’s Estimate, tens of millions of patients undergo general anesthesia annually all over the world [1]. Both the recovery profile and prognosis after surgical anesthesia have gradually become critical indicators for the efficacy and quality of anesthesia [2]. Anesthetic recovery is defined as the state that patients are conscious, can easily be awakened, clearly identify the surroundings and their own identities [3, 4]. Recovery quality focuses mainly on the recovery of consciousness; duration of stay in PACU; and various adverse sequelae,such as pain, PONV, and confusion. The satisfactory quality of recovery from general anesthesia is significant to avoid adverse reactions, such as tachycardia, airway obstruction, hypoxemia, blood pressure fluctuations, and arrhythmia. It remains one big challenge to anesthesiologists to provide a satisfactory quality of recovery for patients, including smooth and rapid extubation and minimal time to resume daily activities. The delayed awakening from anesthesia in clinical practice is still common. It is reported that delayed time could be 40 min in 60% of patients [3]. In most cases, the delayed awakening from anesthesia can be attributed to the residual action of one or more anesthetic and analgesic agents, or adjuvants used in the peri-operative period. Opioids, analgesic agents, are always associated with a number of side effects, including PONV, hyperalgesia, delirium, dysphoria, and respiratory depression [5]. Nalmefene, which is unique as an antagonist against all three types of opioid receptors (µ,κ, and δ), has been used successfully to relieve opioid overdose and reverse postoperative opioid actions, including respiratory depression, sedation, and hypotension, with no impact in postoperative analgesia [6–9]. Nalmefene also has a protective role in cognitive ability of aged patients experiencing video-assisted thoracic surgery and decreases the incidence of delayed neurocognitive recovery [10]. Nalmefene can effectively reduce the injury caused by lung ischemia–reperfusion [11]. But it is obviously insufficient to get a conclusion on the efficacy of nalmefene in optimizing anesthesia recovery from previous studies. A large scale, well-designed and comprehensive clinical study is now urgently needed.

### Aims and objectives

We aim to investigate the effects of nalmefene and provide reliable clinical data on optimizing the recovery quality of patients from general anesthesia. We hypothesize that nalmefene could obviously shorten the time intervals between the end of anesthesia to recovery endpoints (Aldrete recovery score ≥ 9).

### Trial design

This prospective, placebo-controlled, two-arm parallel groups, multicentre, double-blind, randomized, superiority clinical trial was designed to study the efficacy of nalmefene on the recovery quality of patients. Considering the operative time and intraoperative opioid use, patients undergoing moderate-risk surgery will be more representative and first selected, and considering the surgery characteristics of these hospitals, the trial will focus on orthopedic, urologic, and thoracic elective surgery. This study was presented according to the recommendations of Standard Protocol Items: Recommendations for Interventional Trials (SPIRIT) (checklist in the supplemental file, SPIRIT checklist). The study was registered on the ClinicalTrials.gov (No. NCT04713358) on January 19, 2021. We presented the trial registration data in the form of supplemental data.

### Study setting

This study will be carried out in Renji Hospital Shanghai Jiaotong University, School of Medicine; Shanghai Tongren Hospital; First Affiliated Hospital of Guangxi Medical University; and First Affiliated Hospital of Zhengzhou University. This clinical trial has been approved and supported by the ethics committee of Renji Hospital, Shanghai Jiaotong University, School of Medicine (KY2020-150); Shanghai Tongren Hospital (2021–030-01); First Affiliated Hospital of Guangxi Medical University (2021–032); and First Affiliated Hospital of Zhengzhou University (2021-KY-0495–003). The reasons for selection of these hospitals are: First, all of them are large university hospitals, and there are many types of surgery. Second, anesthesiologists in these hospitals have rich experience in clinical researches and are interested in study of the efficacy of nalmefene on patients undergoing anaesthesia. All researchers listed in this protocol have willingness to cooperate to finish this trial.

### Eligibility criteria

Patients will be primarily recruited from our four centers. Table 1 presents a summary of the inclusion and exclusion criteria.

**Table 1:** Inclusion and exclusion criteria for participants

Inclusion Criteria	Exclusion Criteria
1. 18–65 years old 2. Scheduled for orthopedic, urologic, and thoracic elective surgery under general anesthesia with tracheal intubation 3. Physical status I or II of American Society of Anesthesia 4. BMI (kg/m2) ≥ 18 and ≤ 30 5. Estimated anesthesia time 1–4 h 6. Use narcotic analgesics (sufentanil or remifentanil) intraoperatively 7. Use electronic intravenous analgesia pump after surgery 8. Informed consent	1. Refused to participate in this study 2. Refused to receive intravenous analgesia 3. A medical or family history of cognitive disorders, delirium, epilepsy, alienation, anxiety, or depression 4. Recently used anticholinergic drugs, antidepressants, anxiolytics, or anticonvulsants 5. A medical history of organic brain diseases or craniovascular diseases 6. A history of allergy to any drug used in this study 7. A history of drug addiction, alcoholism, or drug abuse 8. Comorbid conditions of severe heart and lung disease; active heart disease; severe hepatic dysfunction ( Child–Pugh class C); severe renal dysfunction ( undergoing dialysis) before surgery; or critical illness (preoperative physical status classification ≥ 3 of American Society of Anesthesia) 9. Participated in other clinical trials within 4 weeks 10. Intraoperative complications, such as cerebrovascular accident, heart failure, or pneumothorax, or transfer to the intensive care unit during hospitalization 11. Unable to communicate in the preoperative period because of coma, profound dementia, language barrier, or incapacity from severe disease 12. Anesthesia time < 1 or > 4 h 13. Chronic pain, which is defined as unsatisfied pain control for at least one month

### Recruitment and consent form

The patients scheduled for elective orthopedic, urologic, and thoracic surgeries under general anesthesia will be candidates of this study. Candidates who meet all inclusion criteria and none of the exclusion criteria in Table 1 will be introduced to participate in this trial. Considering the operative time and intraoperative opioid use, patients undergoing moderate-risk surgery will be more representative and first selected. Designated doctors will explain this trial in detail to our potential participants and provide them with a written informed consent. Participants will have at least 24 h to decide whether they really want to participate in this study or not. Informed consent signed by participants and the patients’ basic data will be obtained before randomization. Moreover, participants will be encouraged to contact the research team if they have any health concerns during trial. The recruitment and obtaining of informed consent by our research members is in line with good clinical practice (GCP). During clinical trial, the researchers should immediately report any serious adverse events, whether related to drugs under study or not, to the director in charge of clinical trial at the research institutions and contact Professor Diansan Su. The steps of procedure in this trial are mainly included in Table 2.

**Table 2:** Schedule of the major study events

	Screening period	Intervention and observation period				
	-7 ~ -1 day	Preoperative	Intraoperative	Sugery ended	Duration of stay in PACU	24 h postoperatively
Informed consent	X					
Medical history	X					
Physical examination	X					
Demographics	X					
ECG	X					X
Blood cell test^a^	X					X
Liver and kidney function^b^	X					
Inclusion /exclusion criteria		X				
Randomization		X				
Intraoperative narcotic analgesics			X			
Non-steroidal antiinflammatory drugs(NSAIDs)			X			
Trial drug				X		
Neostigmine					X	
Atropine					X	
Electronic intravenous analgesia pump				X	X	X
Extubation time					X	
Aldrete score					X	
VAS score					X	X
RASS score					X	
Montreal orientation score					X	
Remedial analgesic sufentanil^c^					X	
PONV					X	X
Pruritus					X	X
Combination medication^d^	X	X	X	X	X	X
Adverse events	X	X	X	X	X	X

Remarks: Collect the latest laboratory and auxiliary examination results within 7 days before the baseline

^a^Blood test: red blood cells, white blood cells, platelet counts, hemoglobin

^b^Liver and kidney function: alanine aminotransferase, aspartate aminotransferase, creatinine, blood urea nitrogen

^c^Remedial analgesic: sufentanil

^d^Combination medication: Medication only recorded at the time of adverse event

### Allocation and blinding

The participants will be randomly assigned into either intervention or control group in a 1:1 ratio with a computer-generated random number sequence in Safety analysis set (SAS) 9.4 software (SAS Institute, Cary, NC, USA). Randomization will be stratified with each center and type of operation. After patient’s screening and randomization, group allocation will occur immediately in sealed and number-coded envelopes. The randomized table contents, numbers as well as their relationship with groups will keep secret from subjects during the whole process. Envelopes containing random codes will be generated and distributed to each center. Drugs will be assigned and allocated according to random numbers by an independent nurse (no further involvement in this research). The study drug will be prepared in a total 2 ml volume by mixing normal saline with a drug dose based on corresponding patient’s body weight. The drug packages will have a completely consistent appearance and they cannot be distinguished from each other with human eyes. During the whole study process, the patients, investigators, intraoperative attending anesthesiologists, data collectors, data analyzers, evaluators, and statisticians will be blinded to group allocation. In a medical emergency, investigators are permitted to open the corresponding emergency envelope as a requirement for identification of a patient’s treatment. Then, they must record a justification about this emergency in the patient’s medical record and case report form(CRF). Serious adverse events will be collected and recorded for further analysis at the end of trial, and investigators must explore the correlation between these events and the patient’s healthcare records.

### Study intervention

All patients should not have preoperative medication. They will have a peripheral vein opened venous access on the day of the operation. After measuring pulse oximetry, electrocardiogram, and noninvasive blood pressure suitable, general anesthesia will be induced in patients with midazolam (0.04–0.06 mg/kg), propofol (1–2 mg/kg), rocuronium (0.6–0.8 mg/kg), and sufentanil (0.3–0.5 μg/kg).During the operation, propofol (1–2 μg/mL) and remifentanil (0.1–0.3 μg/kg/min) have to be maintained, sevoflurane MAC 0.6–1.0 and cisatracurium or rocuronium will be given as needed. These anesthesia agents will be adjust for keeping a Bispectral index (BIS) value of 40 to 60. Estimated 30 min before the end of surgery, cisatracurium or rocuronium infusion will be stopped, and intravenous 0.1 μg/kg sufentanil will be given. Before the end of surgery, parecoxib sodium 40 mg will be intravenously administrated. All anesthetic drugs including propofol, remifentanil and sevoflurane will be stopped when surgery is ended. According to protocol requirements, patients in intervention group will receive nalmefene 0.25 μg/kg and same volume of 0.9% NaCl solution in control group. The reasons why we choose same volume of 0.9% NaCl solution in control group is: 1.we aim to investigate the efficacy of nalmefene on optimizing the recovery quality of patients after general anesthesia. However, nalmefene is not used in conventional resuscitation. 2. the properties of 0.9% NaCl solution are consistent with that of nalmefene, which can be double-blind. For patient-controlled intravenous analgesia (PCIA), sufentanil (2- 2.5 μg/kg) and antiemetic drugs diluted to 100 ml with saline should be immediately and intravenously given to patients. Thereafter, patients will be moved to PACU. In PACU, patients will have neostigmine (0.02–0.04 mg/kg) and atropine (0.01 mg/kg) to antagonize the residual neuromuscular block until their recovery of swallowing function. Tracheal extubation will be carried out when patient is conscious, with a respiratory rate above 12 min − 1 and no residual muscle weakness. Ten minutes later, the arterial blood gases will be recorded. If patient has a VAS score ≥ 4, remedial analgesics such as sufentanil will be given again. Postoperative recovery will be mainly assessed by the time interval from the end of operation to Aldrete score ≥ 9 which is defined as the recovery endpoint. In addition, the time interval from end of operation to extubation, RASS score at extubation, the time at MoCA orientation score ≥ 5, the times and dosages of remedial analgesic sufentanil used in PACU, VAS pain score, and adverse effects will also be recorded. Adherence to interventions primarily refers to clinical investigators adherence.

### Outcomes

#### Primary outcome

The primary outcome is the time interval between the end of anesthesia and Aldrete recovery score ≥ 9 achieved in PACU.

### Secondary outcomes

- 1. The time interval from end of operation to extubation in PACU
- 2. RASS score at extubation
- 3. The time at MoCA orientation score ≥ 5
- 4. The times and dosages of remedial analgesic drug (sufentanil).
- needed in PACU
- 5. VAS pain score at recovery endpoint, and at 1 and 24 h points Post-operatively
- 6. Adverse effects, including PONV and pruritus in PACU
- 7. Incidence of PONV and pruritus within 24 h postoperatively.

### Participant timeline

Table 1 presents the schedule for screening, interventions, assessments and observation for participants. In indexes for the quality of recovery, Aldrete score (0–10) will be divided into five parts: movement, breathing, blood pressure, consciousness, and SpO2, with each part graded as 0, 1, or 2. Higher scores indicate better awakening situation. It will be assessed by each evaluator independently according to the guidelines shown in Table 3.

**Table 3:** The standards for modified Aldrete scores

Items	Standards	Scores
Movement	Moving arms, legs and head spontaneously or by request;	2
	Moving arms or legs spontaneously or by request, restrictedly raising head spontaneously or by request;	1
	Not able to move limbs or raise head	0
Breathing	Deep breathing and effective coughing, normal respiratory rate and amplitude;	2
	Breathing is difficult or restricted, but spontaneous breathing is shallow and slow, and it is possible to breathe through oropharyngeal airway;	1
	Breathing is paused or weak, it requires a respirator therapy or assisted breathing	0
Blood pressure	Within ± 20% before anesthesia;	2
	± 20–49% before anesthesia;	1
	Above ± 50% before anesthesia	0
Consciousness	Completely awakening, answer questions accurately;	2
	Able to wake up, drowsiness;	1
	No reaction	0
SpO2	Air breathing SpO2 > 92%;	2
	Oxygen breathing SpO2 > 92%;	1
	Oxygen breathing SpO2 < 92%	0

Of several scales proposed in literature, we choose RASS [12, 13] to evaluate the incidence and severity of emergence agitation or sedation and classify agitation, sedation, and delirium following general anesthesia. Patients will be divided into two categories based on agitation and sedation levels: non-agitated condition with RASS scores − 5 to 0 and agitated condition 1 to 4. Each evaluator scores the RASS all alone based on the directions shown in Table 4.

**Table 4:** The description of Richmond Agitation-Sedation Scales (RASS)

Score	Term	Description
+ 4	Combative	Overtly combative or violent; immediate danger to staff
+ 3	Very agitated	Pulls on or removes tube(s) or catheter(s) or has aggressive behavior toward staff
+ 2	Agitated	Frequent non-purposeful movement or patient-ventillator dyssynchrony
+ 1	Restless	Anxious or apprehensive, but movements not aggressive or vigorous
0	Alert and calm	
-1	Drowsy	Not fully alert, but has sustained awakening, with eye contact, to voice
-2	Light sedation	Briefly awakens with eye contact to voice
-3	Moderate sedation	Sedation any movement to voice
-4	Deep sedation	No response to voice, but any movement to physical stimulation
-5	Unarousable	No response to voice or physical stimulation

MoCA is a more recently developed cognitive screening tool and has gained worldwide uses in clinical research. It targets the differentiation between the normal aging and mild cognitive impairment (MCI) with a highest point 30, the higher meaning better performance. MoCA contains the following cognitive abilities: visuospatial/ executive function, naming, episodic memory, attention, language, abstraction, and orientation [14]. The orientation score shown in Table 5 is only selected in this study, and include 6 tasks with each correct answer sore 1, the higher meaning better performance.

**Table 5:** MoCA Orientation Score

Orientation						
TASKS	Date	Month	Year	What day	Location	City
Scores	1	1	1	1	1	1

VAS pain score will be judged by an anesthesiologist, who is unaware of the treatment allocation of the institutes, at various stages of recovery, with 10 being possible maximum pain and 0 absent. PONV and pruritus will be estimated by the number of episodes and maybe recorded as a 0–10 numerical rating score.

### Sample size

The plan of sample size is mainly based on the time interval between the end of anesthesia and Aldrete recovery score ≥ 9. At present, this time interval reported in literature is controversial from 70–83 [15] to 25–40 min [16]. Trescot AM’s study showed that complete physiologic recovery takes place by 40 min in 40% of patients [3]. In our previous research, we found that the average time interval from the end of anesthesia to Aldrete score ≥ 9 without nalmefene is 30 ± 30 min. With the use of nalmefene for patients undergoing elective moderate-risk surgery, the time interval is assumed to be shortened to about 20 min. The total type I error was α = 0.05. The sample size was calculated by a two-sided test with 1:1 randomization using PASS 19 software (Informer Technologies, Inc. Beijing, China). There are 235 individuals estimated for each group (total of 470 cases). Considering that the sample shedding rate is usually about 10%, the final sample size is planned to be 520. The enrolled patients will be randomized and equally divided into the intervention group (*n* = 260) and control group (*n* = 260).

### Ethics and Dissemination

#### Plans for communicating important protocol modifications to relevant parties

All changes to this protocol will be reviewed and proved by the ethics committee and will be reported to the sponsor, participating care providers, and investigators. Composition, role, and reporting structure of the data monitoring.

### Committee

Nalmefene is very safe and easy controlled in clinical practice. Therefore, it is not neccery to setup the data monitoring.

### Interim analyses

No plan on formal interim analyses in this study.

### Frequency and plans for auditing trial conduct

In this investigator-initiated pragmatic trial, we have no plan for auditing trial conduct. A special staff from the clinical research center of Renji Hospital would monitor the whole procedure of thetrial. Due to limited funds, no additional contract research organization was invited.

### Data management

All patient data collected from this clinical trial will be recorded and/ or filed in the appropriate CRF. These data include study number, subject number, date submitted subject information, informed consent, and each visit date. Source data should be submitted in accordance with GCP guidelines. The data manager will be in charge of data processing according to sponsor’s standard operating procedure. At same time, regular monitoring will be conducted to ensure that data adequacy, accuracy, and completeness.Once the quality assurance procedure completed, the database will be locked. The principal investigator will review the data in our trial. In addition, we have a special staff from the clinical research center of Renji Hospital to check the data quality of the trial regularly, and the scientific research department of the hospital will archive the data finally. The participants data will be kept 5 years at least. Participant information will be confidential and managed according to the Data Protection Act, NHS Caldecott Principles, The Research Governance Framework for Health and Social Care, and the conditions of Research Ethics Committee approval.

### Statistical analysis

This study will use the following:

- 1.Full Analysis Set (FAS), which is an ideal set of subjects and in accordance with the principle of intention-to-treat analysis. This dataset is derived from all randomized subjects, with minimal and reasonable exclusion. When FAS is selected for statistical analysis, the estimation of the missing value of main index value can be done by the nearest observation.
- 2.Per-protocol Set (PPS), which is a subset of FAS. These subjects are more related to protocol, including considerations such as treatment, the feasibility of outcome measures, and the absence of major protocol violations. The rationale for excluding subjects from PPS should be stated at the time of blinded review and documented prior to unblinding.
- 3.SAS, which analyzes the population safety from all subjects who received at least one dose of treatment. Analysis of safety data in the study will be based on SAS.

### Statistical methods for the primary outcome

The main analyses for primary outcome will be performed based on FAS population. The description for the time interval from the end of anesthesia to Aldrete score ≥ 9 in PACU will be reported as mean (SD) for two groups. The primary outcome will be analyzed and calculated using a general linear model (GLM), with center variable as random effect. The time interval difference and 95% confidence interval (CI) between intervention group and placebo group will be calculated. The primary outcome will be considered as achieved when the two-sided *p*-value is less than 0.05 and the upper boundary of 95% CI is less than zero. The primary analysis has been performed among the FAS population without missing outcome data. We do not anticipate any missing data due to a short time frame for primary outcome. Nevertheless, we will use multiple imputations to handle substantial missing data for sensitivity analyses. The additional sensitivity analyses will also be conducted among the PPS population.

### Statistical methods for the secondary outcomes

These analyses will be performed on FAS and PPS populations. The t-test or Wilcoxon rank sum test will be used to make comparison of time interval from the end of operation to extubation and to Montreal score ≥ 5 in PACU between two groups. The Wilcoxon rank sum test will also be used to compare RASS score and VAS pain score between groups. The comparison of percentage from patients requiring remedial analgesic drug will be done between groups with chi-square test. For safety outcomes from SAS population, such as incidence of PONV, the chi-square test will also be used for their comparison between groups.

### Description of baseline characteristics

In this study, the description for baseline characteristics will be performed on the FAS population and compared between two randomized groups. All demographics and baseline characteristics will be described in appropriate summary statistics, such as frequency and proportion for dichotomous data, mean and standard deviation (SD) for continuous data, or median and interquartile range, if more appropriate,for continuous data.

### Subgroup analyses

We will also explore whether outcomes are different between groups, in terms of baseline variables, such as age, gender, site, etc.

### Software details

All analytic data will come from SAS, version 9.4 or above (SAS Institute, Cary, NC, USA). Figures will be drawn with R software, version 4.1.2 or above (Free Software Foundation, Inc. Boston, MA, USA).

### Patient and public involvement

Patients and/or the public were not involved in the design, conduct, reporting, or dissemination plans of this research. By obtaining an consent form, clarifying the risks associated with anesthesia, the patient will be informed of the trial. They will be told that the effect of nalmefene may be improved the quality of recovery. Since they are blinded to the study, they will not know which group they are assigned to. If it is for personal benefit, each study participant can contact the main researcher conducting anesthesia and ask about the results of the trial after the database is officially closed and the data is analyzed.

### Ethics, dissemination and safety monitoring

This study will be conducted in compliance with the protocol, the current version of the Declaration of Helsinki, the ICH-GCP (International Conference for Harmonisation of Technical Requirements for Registration of Pharmaceuticals for Human Use-Good Clinical Practice) guidelines as well as national legal and regulatory requirements. Findings will be disseminated through peer-reviewed publication that will be made publicly available on PubMed Central on acceptance for publication, in compliance with NIH public access policy. The experimental should be terminated in time when the following situations occur:1 Serious adverse events occur, and the adverse events are likely to be related to the trial intervention drugs. At this time,there may be major problems with the drug safety, and the trial should be terminated in time;2 External information (such as other high-quality studies or evidence) proves that the intervention program is ineffective or effective, and there is no need to continue the current clinical trials. Additional consent provisions for collection and use of participant data and biological specimens. No ancillary study is planned, and we have no plans to collect additional participant data or biological specimens for use outside of this protocol.

### Adverse event reporting and harms

Nalmefene is an opiate receptor antagonist and used to treat acute opioid overdose and to help in alcohol dependence and addictive behaviors. It is a drug and generally well tolerated, but it may cause some minor adverse effects. We will evaluate any abnormal reactions from our participants whatever they are from nalmefene. In the study-specific CRF, there is also a commentary section, in which our members will report all allocation violations or unexpected side effects from the clinical process. We will collect and report all expected and unexpected drug-related adverse events in the trial publications.

### Provisions for post-trial care

Participants will get free treatment if they are injured and in case of damage related to our clinical research work.

## Discussion

To our knowledge, this study is the first large-scale PPPMDR clinical trial to study the efficacy of nalmefene to upgrade anesthetic recovery quality. The concept of anesthetic recovery is based on the termination of anesthetic effect rather than regaining of consciousness. The Aldrete score has been applied in PACU to assess the recovery quality of patients from anesthetic experience and serves as a basis to discharge patients from PACU whether to transfer to hospital ward or send home [15]. Castro reported that Aldrete score in ≥ 9 could reflect the stability of patient’s vital signs, regaining consciousness, protective reflexes, and muscular activity [17]. The time interval for patients from the end of anesthesia to Aldrete score in ≥ 9 varies a lot among different hospitals. A randomized comparison study concluded that the time interval between the end of anesthesia and Aldrete score ≥ 9 is 7 –83 min [15]. But another trial found it to be 25–40 min [16]. And what’s more, Frost reported that complete physiologic recovery took place by 40 min in 40% of patients, and even a completely recovery of functional quality was in 11% on day 3 [3]. Nevertheless, Zelcer reported that incidence of unresponsiveness for anesthesia-related recovery between 15 and 90 min is 9% [18]. Mechanical ventilation is always associated with severe complications with time-dependent complications. A prolonged duration of intubation is often the main reason for a higher incidence of complications, including ventilator-associated pneumonia (VAP), and increased mortality [19]. In our primary research, the time interval between the end of anesthesia and Aldrete recovery score ≥ 9 could be reduced to 20 min with nalmefene for patients undergoing elective moderate-risk surgery. Based on the premise of ensuring patient’s safety and comfort, nalmefene can also short mechanical ventilation time, stay time in PACU, and finally improve efficacy of anesthetic-related recovery. Delayed awakening or emergence from anesthesia can be attributed to complicated drug residues including benzodiazepines (BDZs), opioids, inhalation anesthetics, neuromuscular blockade agents (NMBA), and adjuvants. Although opioids remain the cornerstone of anesthesia and pain therapy, they are more difficult to control than neuromuscular relaxants or benzodiazepine in clinical experience. Opioids produce many effects by µ receptors through engaging two important signaling pathways: the G protein and β-arrestin pathways. The former pathway results in analgesia, whereas the latter one in opioid side- effects including respiratory depression, PONV, pruritus, dependence, dysphoria, and sedation [20]. κ receptors are responsible for spinal analgesia, sedation, delirium, dyspnea, and respiratory depression [5]. Residual effect of opioids is one of common reasons for delayed awakening because opiods can act on both µ and κ receptors. Nalmefene was approved by FDA in 1995 as an opioid antagonist. Since then, it has been widely used in clinic to reverse opioid effects including respiratory depression and opioid overdose by inhibiting G protein and β-arrestin recruitment in brain cortex and striatum [21–23]. In addition, it could also reverse the respiratory depression from opioid overdose [24]. In comparison with other opioid receptor antagonists, it was found that nalmefene has the highest affinity to μ opioid receptors in rat; similar affinity to μ and κ receptors, but much lower affinity to δ receptors in humans [25, 26]. And it differs from other opioid antagonists by its more long-lasting µ- opioid receptor blockade without intravenous repetition, higher bioavailability, and lower liver toxicity. Nalmefene hydrochloride can effectively inhibit the signal pathway of TLR4, and can effectively reduce the injury caused by lung ischemia–reperfusion. The large dose is closely related to the good effect, which is worthy of promotion [11]. Li’s study shower that nalmefene could also significantly ameliorate decline in memory ability, performance function and promote neurocognitive recovery [10]. In clinic, nalmefene in low-dose could effectively reverse opioid-induced side effects and optimize anesthetic recovery quality with less adverse reactions in both children and adults [27]. In a single dose of 0.25 µg/kg, it could improve the analgesia effect for postoperative pain control and decrease opioid side effects including PONV and pruritus [9, 21, 28]. However, it is still insufficient on previous studies of nalmefene, such as the sample size in each research was not large enough to represent all cases in clinic, the time and dosage for nalmefene administrated to patients were not fixed, and the data about that nalmefene promotes anesthetic recovery quality and cut down the adverse reactions is not in detail. At present, whether the use of nalmefene can short the recovery time after general anesthesia and reduce the delayed awakening of patients needs to be settled. So, It is definitely necessary to have a large group of sample, and PPPMDR clinical trial to study and evaluate the safety and efficacy of nalmefene with regard to optimize recovery quality and reduce the adverse effects after general anesthesia. That is just what this protocol plans to do. In this protocol, our goal is to provide a clinical procedure with nalmefene to achieve a satisfactory anesthetic recovery including both smooth and rapid extubation, satisfactory analgesia, and minimal incidence of opioid side effects. We have chosen Aldrete, RASS and Montreal score, which are commonly used as clinical scales, to evaluate the quality of anesthetic recovery. It has been validated that RASS can detect changes in sedation status and set against constructs in terms of level of consciousness and delirium [29]. MoCA orientation is a simple method which can do cognitive screening in stand-alone with superior sensitivity [30]. Based on the above and other medical measures selected and modified in this protocol, we believe that our goal will certainly be achieved by the united efforts of our team members.

### Trial Status

This trial was registered at CliniclTrials.gov, NCT044713358 on January 19,2021. Protocol version 1.1 was approved in December 2020. This study started in September 2021, and the recruitment phase will last until December 2023.

## Data Availability

Data on the patients cannot be made publicly available because of Chinese data protection rules and regulations. The statistical code will be available upon request.
